# Time use in out-of-class activities and its association with self-efficacy and perceived stress: data from second-year medical students in China

**DOI:** 10.1080/10872981.2020.1759868

**Published:** 2020-05-18

**Authors:** Xinzhi Song, Ning Ding, Nan Jiang, Honghe Li, Deliang Wen

**Affiliations:** Institute for International Health Professions Education and Research, China Medical University, Shenyang, China

**Keywords:** Time use, self-efficacy, perceived stress, medical students

## Abstract

**Background**: Medical students struggle with heavy workloads and face unique time management challenges, calling for insight on medical students’ time use in out-of-class activities and related psychological factors.

**Objectives**: To investigate Chinese medical students’ time use in out-of-class activities and its association with self-efficacy and perceived stress.

**Design**: 940 second-year medical students were invited to participate in the survey. A questionnaire was developed to investigate the amount of time participants spent on out-of-class activities. Two existing instruments were used to assess participants’ self-efficacy and perceived stress. Descriptive statistics and logistic regression were used to analyze the data.

**Results**: 686 students (72.98%) completed the survey. On average, students spent about 14.4 hours per week (HPW) preparing for class and studying, 13.8 HPW on leisure and recreation, 4.8 HPW on physical exercise, 4.1 HPW on student clubs, 1.9 HPW on volunteering, and 1.9 HPW on part-time jobs on/off-campus. After controlling for demographic factors, higher self-efficacy was associated with more time preparing for class and studying (OR = 1.29, 95%CI: 1.10 ~ 1.52). Higher perceived stress was associated with less time preparing for class and studying (OR = 0.84, 95%CI: 0.72 ~ 0.98), exercising (OR = 0.82, 95%CI: 0.69 ~ 0.97), and volunteering (OR = 0.81, 95%CI: 0.67 ~ 0.97).

**Conclusions**: Chinese medical students reported more time spent on preparing for class and studying and less time spent on volunteering and part-time jobs on/off-campus. Time spent preparing for class and studying was associated with higher self-efficacy and lower perceived stress, while perceived stress was also associated with less time spent on physical exercise and volunteering. Medical schools may need to employ measures to develop their students’ self-efficacy and maintain a relatively low level of perceived stress, especially in students who also have a low level of self-efficacy.

## Introduction

Out-of-class activities are a natural and essential part of the college experience, and how college students use their time in these activities has important implications for students’ level of engagement, learning, and development [1–4]. Kuh et al. have stated, ‘what students do during college counts more in terms of desired outcomes than who they are or where they go to college’ [5]. Astin’s theory of student involvement asserted that the activities a student engages in outside the classroom (e.g. preparing for class and studying, clubs, sports, and recreation) can have a positive impact on academic learning and personal development as long as he or she is oriented toward enhancing the college experience [6]. Other studies have indicated that with increased time in extracurricular activities, students’ social and academic adjustment, educational involvement, academic achievement, life management, interpersonal skills, social adaption and cooperativeness, and career development skills can all increase [7–10].

The importance for understanding time use in out-of-class activities among students of tertiary education institutions has been highlighted, but it still proves to be a surprisingly underexplored concept. Available data on time use patterns of college students have suggested that undergraduates’ time use patterns have changed over time [11]. To further explore the concept of time use and its patterns, Brint and Cantwell have developed a measure conceptualizing the different dimensions of time spent in/out of class in order to investigate the time usage of undergraduate students of the University of California [12]. These dimensions include ‘scholarly’ time use (e.g. attending class, preparing for class), ‘active’ time use (e.g. physical exercise, volunteering), ‘passive’ time use (e.g. watching television, use of computer for fun), ‘connecting’ time use (e.g. paid employment on campus, student clubs), and ‘separating’ time use (e.g. paid employment off-campus, religious activities). Findings showed that students high on active and low on passive time use tended to be more conscientious of their academic work than students high on connecting and separating time use. On average, students tended to spend equal time on academics and on leisure, but those who spent more time in leisure and off-campus jobs had poorer academic performance and personal ability development.

Medical schools are a distinct part of tertiary education institutions. Medical students struggle with a heavy workload during their comparatively long course of study [13], and they face unique time management challenges. Understanding time use in the undergraduate medical education setting would help point the way to improving time management skills in medical students, as good use of time enables medical students to prioritize and balance their studies with other aspects of their life. In fact, evidence shows that good management of the limited resource of time helps to reduce stress in medical school and contributes to the academic performance and personal development of medical students [14]. Furthermore, these outcomes are directly related to whether a medical student can become a qualified medical practitioner [15]. These findings suggested that, to improve academic performance and personal competency, understanding how students use their time in medical school would help institutions arrange for activities that could not only enhance medical students’ time management skills but also their overall undergraduate medical experience.

Much attention has been paid to students’ time use and its association with their academic performance and personal development [16–18]. However, to our knowledge, few studies have focused on time use and its related psychological factors. Studies have shown that self-efficacy and perceived stress, as important psychological variables, can affect an individual’s attitude, behavior, and emotional state towards problems, which might affect medical students’ learning, physical exercise, and other behaviors [19,20]. Self-efficacy, one of the constructs of Bandura’s Social Cognitive Theory, is defined as ‘one’s belief of his/her abilities to mobilize the motivation, cognitive resources, and courses of action needed to achieve an intended goal in a specific life situation’, which can be generally stated as ‘I can because I believe I can’ [21]. Self-efficacy is believed to be important for motivation to take action and to support the effectiveness of various undertaken activities [19]. Perceived stress refers to ‘a condition or feeling experienced when an individual perceives that the demands placed on them exceed the resources the individual has available to meet that demand’ [22]. High levels of perceived stress can cause psychosocial consequences, such as increased levels of depression and anxiety as well as burnout and reduced quality of life [23], all of which may hinder students’ active engagement in extracurricular activities.

The purpose of this study was to investigate the status of medical students’ time use in out-of-class activities and the potential association of self-efficacy, perceived stress, and their interaction on time use in out-of-class activities. Results would help medical schools build extracurricular activities that may improve time management skills in students and also address certain psychological variables, such as self-efficacy and perceived stress.

## Methods

### Design and sampling

This cross-sectional study was conducted via cluster sampling on all 940 second-year students majoring in clinical medicine at China Medical University in Shenyang, Liaoning province during December 2018. The questionnaires were distributed through Sojump (an online survey platform). Participants completed and submitted the questionnaire online and were able to withdraw from the survey at any time. All students who participated in the survey signed the informed consent form. The study protocol and questionnaire were approved by the China Medical University Ethics Committee.

## Measures

### Demographics

A demographic questionnaire was used to collect information on participants’ age, sex, household registration area (urban/rural), birthplace, duration of medical program, and parents’ education levels, occupation, and annual household income. The household registration area (urban or rural) is an assigned residence registration system for all Chinese residents upon birth or attaining citizenship [24]. Durations of medical programs usually comprise two major streams: a 5-year bachelor degree program or an 8-year bachelor and master’s combined program [25]. Based on available literature, these demographic variables were selected for two primary reasons. First, participants’ personal characteristics may lead to differential constraints on time use. For instance, female students were expected to be more diligent in their studies and may therefore devote more time to preparing for class and studying [18] while also devoting less time to physical exercise than male students [26]. Second, participants’ family characteristics were expected to influence their preferences. For example, students from higher socio-economic backgrounds may devote more time to leisure activities as they have a financial safety net from their parents [27]. All these demographic variables were controlled to limit confounders and better explain the association between time use in out-of-class activities and self-efficacy and perceived stress.

### Time use in out-of-class activities

A questionnaire for time use in out-of-class activities was developed in reference to the NSSE (National Survey of Student Engagement) Survey Instrument 2018 Version [28] and was used to investigate the amount of time spent on out-of-class activities among Chinese medical students. These categories specifically focused on the allocation of time to six types of activities: preparing for class and studying (reading, writing, doing homework or lab work, etc.), volunteering (community service, etc.), physical exercise (sports, fitness, etc.), student clubs (calligraphy, singing, etc.), part-time jobs on/off-campus, and leisure and recreation (watching TV, video games, shopping, etc.). All the students who participated in the survey reported time use in seven discrete ranges of hours per week (HPW) (1 = 0 h, 2 = 1–5 h, 3 = 6–10 h, 4 = 11–15 h, 5 = 16–20 h, 6 = 21–25 h, 7 = 26 h or more). Time between ranges (e.g. 5.5 h) would be rounded to the next range (e.g. 3 = 6–10 h). Fosnacht’s research on student time use in college was used a reference when calculating mean time use [1], so discrete ranges were recoded to their midpoint values, and the unbounded upper choice of ‘26 h or more’ was assigned a fixed value of ‘28 h’ (1 = 0 h, 2 = 3 h, 3 = 8 h, 4 = 13 h, 5 = 18 h, 6 = 23 h, 7 = 28 h). Time use data was captured in ranges and recoded to point estimates so that the data would not be viewed as precise estimates of student time use.

### Self-efficacy

The General Self-Efficacy Scale (GSES) was used to assess students’ self-efficacy, which was defined as one’s belief of the possibility of achieving an intended goal in specific life situations [21]. The GSES consisted of 10 items scored on a 4-point Likert scale, and participants’ responses ranged from 1: ‘not at all true’ to 4: ‘exactly true’, with total scores ranging between 10 and 40. A higher score indicated higher self-efficacy. The revised Chinese version of the scale had demonstrated good reliability and sufficient validity evidence [29]. Cronbach’s α was computed for the use of the GSES in this study.

### Perceived stress

The Perceived Stress Scale (PSS) is a self-reported instrument used to evaluate perceived daily stress in the past month [30]. There are three versions of the perceived stress scale at present: the 14-item scale (PSS-14), the 10-item scale (PSS-10), and the 4-item scale (PSS-4), which were all compiled by Cohen et al [31]. Psychometric properties of the PSS-10 were found to be superior to those of the PSS-14 and PSS-4 [30], so the PSS-10 was used in this study. The PSS-10 consisted of six negative items and four positive items rated on a 5-point Likert scale ranging from 0: ‘never’ to 4: ‘very often’. The total score was the sum of all items scores, with scores ranging between 0 and 40. A higher score indicated higher perceived stress. The revised Chinese version of the scale had demonstrated good reliability and extensive validity evidence among Chinese undergraduates [32]. Cronbach’s α was computed for the use of the PSS-10 in the study.

### Statistical analysis

A descriptive analysis was performed, and values were reported as mean ± standard deviation (SD) for continuous variables and as frequencies and percentages for categorical variables. Binary logistic regression or ordinal logistic regression (selected only if the ordinal regression model passed the parallel lines test) was performed to examine the associations between dependent variables (time use in the six categories of out-of-class activities) and independent variables (self-efficacy, perceived stress, and their interaction term).

For standardization of measurement scales during statistical analysis, Z scores were computed for the independent variables of self-efficacy and perceived stress. The interaction term between self-efficacy and perceived stress was examined for statistical significance using the Wald test. A *p* value was calculated to decide whether or not to accept the use of the model with the interaction term.

Covariates included personal characteristics (age, sex, household registration area, birthplace, duration of medical program) and family characteristics (parents’ education level, parents’ occupation, annual household income). Due to the fact that Chinese mothers have much higher participation in their children’s education than fathers, only maternal characteristic variables were controlled in the statistical analysis [33]. To account for all missing data of covariates, multiple imputation (MI) by chained equations were carried out. Ten imputed datasets were generated.

All variables were simultaneously entered into each of the regression models. Odds ratios (OR) and 95% confidence intervals (95% CI) were estimated for all variables. A two-tail *p* value <0.05 was considered to be statistically significant. All analyses were carried out using STATA 13.0 (Stata Corp., College Station, TX, USA).

## Results

### Participant and instrument characteristics

Of the 940 medical students invited to participate in this study, 686 (72.98%) effectively completed and returned the questionnaire. The rate of missing data for demographic variables was less than 5%, and there were no missing data for the GSES or the PSS-10. Mean GSES and PSS-10 scores were 25.43 ± 6.11 and 16.29 ± 5.71, respectively. Cronbach’s alphas for the GSES and PSS-10 were 0.94 and 0.85, respectively. Table 1 summarizes the characteristics of the participants and instruments, including the number of corresponding missing values.

**Table 1.** Characteristics of the second-year medical students enrolled in the study (n = 686)

Variable	
Self-efficacy, mean±SD	25.50 ± 6.21
Perceived stress, mean±SD	16.30 ± 5.68
Demographic factors	
Age, mean±SD	20.27 ± 0.73
Sex, n(%)	
Male	276(40.23)
Female	410(59.77)
Household registration area, n(%)	
Rural	220(32.07)
Urban	453(66.03)
Missing	13(1.90)
Birthplace, n(%)	
Liaoning province	358(52.18)
Other provinces	325(47.38)
Missing	3(0.44)
Duration of medical program, n(%)	
Five years	521(75.95)
Eight years	165(24.05)
Mother’s education level, n(%)	
No high school	245(35.71)
High school	199(29.01)
Post-secondary or higher	236(34.40)
Missing	6(0.87)
Mother’s Occupation, n(%)	
Legislators, senior officials, and managers	96(13.99)
Professionals, technicians, and associate professionals	89(12.97)
Clerical support workers	69(10.06)
Service and sales workers	118(17.20)
Skilled agricultural, forestry, and fishery workers	82(11.95)
Craft and related trades workers, plant and machine operators and assemblers	22(3.21)
Other	202(29.45)
Missing	8(1.17)
Annual household income, n(%)	
<¥20,000	150(21.87)
¥20,000~¥50,000	163(23.76)
¥50,000~¥100,000	307(44.75)
>¥100,000	45(6.56)
Missing	21(3.06)

### Distribution of time in out-of-class activities

On average, participants spent about 14.4 h per week (HPW) on preparing for class and studying, 13.8 HPW on leisure and recreation, 4.8 HPW on physical exercise, 4.1 HPW on student clubs, 1.9 HPW on volunteering, and 1.9 HPW on part-time jobs on/off-campus (Table 2). More than half (54.53%) of the students reported that they did not spend any time volunteering. A majority of medical students reported that they did not spend any time on part-time jobs either on or off-campus (82.51% and 87.61%, respectively).

**Table 2.** Distribution of time use in out-of-class activities among medical students enrolled in the study (n = 686)

Variable	Time use (h per week)						
	0	1-5	6-10	11-15	16-20	21-25	>25
Preparing for class and studying, n (%)	5 (0.73)	114 (16.62)	144 (20.99)	134 (19.53)	108 (15.74)	76 (11.08)	105 (15.31)
Leisure and recreation, n (%)	9 (1.31)	70 (10.20)	171 (24.93)	195 (28.43)	116 (16.91)	41 (5.98)	84 (12.24)
Student clubs, n (%)	87 (12.97)	477 (69.53)	83 (12.10)	18 (2.62)	6 (0.87)	3 (0.44)	10 (1.46)
Physical exercise, n (%)	77 (11.22)	424 (61.81)	128 (18.66)	30 (4.37)	13 (1.90)	2 (0.29)	12 (1.75)
Volunteering, n (%)	371 (54.08)	276 (40.24)	30 (4.37)	2 (0.29)	2 (0.29)	0 (0)	5 (0.73)
Part-time job on campus, n (%)	566 (82.51)	81 (11.81)	22 (3.21)	10 (1.46)	1 (0.15)	0 (0)	6 (0.87)
Part-time job off campus, n (%)	601 (87.61)	52 (7.58)	19 (2.77)	7 (1.02)	2 (0.29)	1 (0.15)	4 (0.58)

### Ordinal and binary logistic regression analyses for time use in out-of-class activities, self-efficacy, and perceived stress

Five of the 686 participants reported that they did not spend any time on preparing for class and studying, and nine did not report spending any time on leisure and recreation. Therefore, the levels of time use of these two activities were merged and readjusted to 6 ranges instead of the original 7 (1 = 0–5 h, 2 = 6–10 h, 3 = 11–15 h, 4 = 16–20 h, 5 = 21–25 h, 6 = 26 h or more). Less than 10% of the participants spent more than 10 h per week on physical exercise or student clubs, so the level of time use of these two activities were merged and readjusted to 4 ranges (1 = 0 h, 2 = 1–5 h, 3 = 6–10 h, 4 = 11 h or above). Parallel line tests for ordinal logistic regression passed for the following dependent variables: preparing for class and studying (χ^2^ = 12.28, p = 0.66); physical exercise (χ^2^ = 11.91, p = 0.06); student clubs (χ^2^ = 8.95, p = 0.18); and leisure and recreation (χ^2^ = 14.30, p = 0.28).

Time use in volunteering and time use in part-time jobs on/off-campus were recoded as binary variables, ‘Yes’ (constituted by ≥1 h/week) and ‘No’ (constituted by = 0 h/week), for the binary logistic regression model, because more than half of the participants reported that they did not participate in volunteering or in part-time jobs both on and off-campus.

Tables 3-5 show the associations between time use in the different categories of out-of-class activities and self-efficacy, perceived stress and their interaction. All the models, including covariates, are shown in an online supplementary file.

**Table 3.** Associations between time spent on preparing for class and studying and self-efficacy and perceived stress using multivariate ordinal logistic regression (n = 686)

Variable	Preparing for class and studying, OR(95%CI)			
	Model 1 ^a^	Model 2 ^b^	Model 3 ^c^	Model 4 ^d^
Self-efficacy	1.40(1.22–1.62)***	–	1.29(1.10–1.52)**	1.31(1.11–1.53)**
Perceived stress	–	0.75(0.65–0.86)***	0.84(0.72–0.98)*	0.84(0.71–0.98)*
Self-efficacy × Perceived stress	–	–	–	1.04(0.93–1.16)

Note: *p <.05; **p < .01; ***p < .001. ^a^ Only self-efficacy was included; ^b^ Only perceived stress was included; ^c^ Self-efficacy and perceived stress were both included; ^d^ Self-efficacy, perceived stress, and their interaction term were included. Controlled for all covariates in each of the models.

**Table 4.** Associations between time use in out-of-class activities (leisure and recreation, physical exercise, student clubs) and self-efficacy and perceived stress using multivariate ordinal logistic regression (n = 686)

	Leisure and recreation		Physical exercise		Student clubs	
Variable	OR(95%CI) ^a^	OR(95%CI) ^b^	OR(95%CI) ^a^	OR(95%CI) ^b^	OR(95%CI) ^a^	OR(95%CI) ^b^
Self-efficacy	1.08(0.93–1.27)	1.10(0.94–1.29)	1.04(0.87–1.23)	1.10(0.93–1.31)	1.03(0.85–1.24)	1.06(0.87–1.29)
Perceived stress	1.12(0.95–1.32)	1.12(0.95–1.31)	0.81(0.68–0.96)*	0.82(0.69–0.97)*	0.85(0.71–1.03)	0.85(0.71–1.03)
Self-efficacy × Perceived stress	–	1.06(0.95–1.18)	–	1.17(1.04–1.31)**	–	1.10(0.97–1.24)

Note: *p < .05; **p < .01; ***p < .001. ^a^ Self-efficacy and perceived stress were both included; ^b^ Self-efficacy, perceived stress, and their interaction term were included. Controlled for all covariates.

**Table 5.** Associations between time use in out-of-class activities (volunteering, part-time jobs on/off campus) and self-efficacy and perceived stress using binary logistic regression (n = 686)

	Volunteering		Part-time job on campus		Part-time job off campus	
Variable	OR(95%CI) ^a^	OR(95%CI) ^b^	OR(95%CI) ^a^	OR(95%CI) ^b^	OR(95%CI) ^a^	OR(95%CI) ^b^
Self-efficacy	1.15(0.96–1.37)	1.19(0.99–1.43)	0.88(0.70–1.11)	0.91(0.72–1.16)	0.85(0.65–1.11)	0.86(0.65–1.13)
Perceived stress	0.81(0.67–0.97)*	0.80(0.67–0.97)*	1.04(0.82–1.31)	1.06(0.84–1.33)	1.04(0.79–1.35)	1.05(0.80–1.38)
Self-efficacy × Perceived stress	–	1.10(0.97–1.24)	–	1.10(0.93–1.29)	–	1.05(0.87–1.26)

Note: *p < .05; **p < .01; ***p < .001. ^a^ Self-efficacy and perceived stress were both included; ^b^ Self-efficacy, perceived stress, and their interaction term were included. Controlled for all covariates.

#### Time use in preparing for class and studying

Table 3 shows the association between time use in preparing for class and studying and self-efficacy (Model 1), perceived stress (Model 2), both self-efficacy and perceived-stress (Model 3), and the interaction between self-efficacy and perceived stress (Model 4). Each model also included all covariates. Model 3 was selected for analysis because there was no statistically significant interaction between self-efficacy and perceived stress in relation to time use in preparing for class and studying (p = 0.26). After controlling for potential confounding factors, a higher level of self-efficacy was associated with increased odds for more time in preparing for class and studying (OR = 1.29, 95%CI: 1.10 ~ 1.52). A higher level of perceived stress was associated with decreased odds for more time in preparing for class and studying (OR = 0.84, 95%CI: 0.72 ~ 0.98).

#### Time use in physical exercise

**Figure 1.**
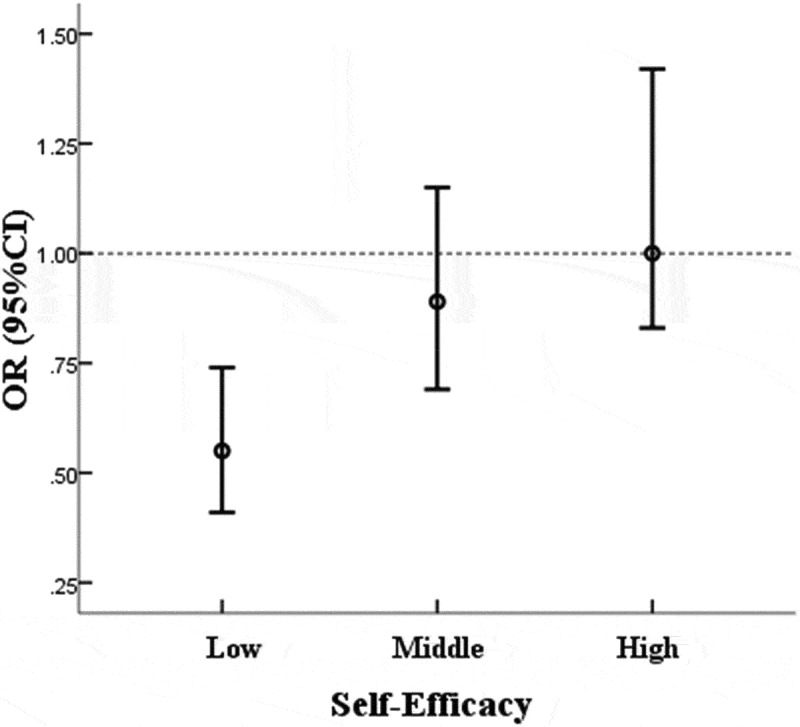
ORs (95%CI) between perceived stress and time spent on physical exercise at different levels of self-efficacy among medical students

The model with the interaction term was selected because the interaction term was statistically significant (p < 0.01) for time spent on physical exercise (Table 4). A higher level of perceived stress was associated with decreased odds for more time spent on physical exercise (OR = 0.82, 95%CI: 0.69 ~ 0.97). The interaction between self-efficacy and perceived stress and its association with time spent on physical exercise was presented using ORs (95%CI) for perceived stress at three different levels of self-efficacy (‘low’ = lower than one SD below the mean; ‘middle’ = between one SD below the mean and one SD above the mean; ‘high’ = higher than one SD above the mean). When self-efficacy increased from the low level to the high level, OR for perceived stress also increased for time spent on physical exercise, changing from lower than 1 (OR = 0.55, 95% CI: 0.41 ~ 0.74) to not statistically significant (Figure 1).

#### Time use in volunteering

The model without the interaction term was selected for analysis because the interaction term was not statistically significant (p = 0.15). Binary logistic regression analysis showed that a higher level of perceived stress was associated with decreased odds for participating in volunteering activities (OR = 0.81, 95%CI: 0.67 ~ 0.97) (Table 5).

#### Time use in leisure and recreation, student clubs, and part-time jobs on/off-campus

There were no statistically significant associations between self-efficacy or perceived stress and time spent on leisure and recreation (p = 0.19), student clubs (p = 0.13), or part-time jobs on/off campus (p = 0.27, p = 0.63) (Tables 4 and 5).

## Discussion

To our knowledge, this is the first study to report the associations between time use in out-of-class activities and self-efficacy and perceived stress. Findings among Chinese medical students showed that self-efficacy had a positive association with time spent on preparing for class and studying, while perceived stress had a negative association with time spent on preparing for class and studying, physical exercise, and volunteering. The interaction term was statistically significant between self-efficacy and perceived stress for time use in physical exercise.

In this study, second-year medical students were selected for the following considerations. The second year of medical school was considered to be a critical period. Students have already experienced a year of medical school life and acquired certain time management skills to adjust to their new learning environment [34]. Therefore, the amount of time used in out-of-class activities would tend to be more stable to some degree. Furthermore, students would be approaching their third year, during which they would be busy with specialized courses and clinical clerkship [35]. Understanding medical students’ time use before they get even busier with clinical studies may better predict how well they would perform in clinical practice. For example, research shows that participating in volunteering activities in pre-clerkship years had a prevention effect, which can deter unethical behaviors among medical students in clinical practice [36]. Of particular note is that Chinese medical students were typically admitted to medical school immediately after high school, so this study can provide practical evidence for countries that offer similar undergraduate medical programs, such as the UK, Australia, and Japan [37].

The results suggested that Chinese medical students spent the most time on preparing for class and studying, followed by leisure and recreation, physical exercise, and student clubs, whereas the least time was spent on volunteering and part-time jobs on/off-campus. These findings are similar to the study by Fosnacht et al. on time use among American college students [1]. Fosnacht et al. used data from 2014 to 2015 administrations of the National Survey of Student Engagement (NSSE) and analyzed first-year students’ time use in college. They found that students spent the most time (about 13.8 HPW) preparing for class, followed by relaxing and socializing (about 12.3 HPW). They also found that students spent about 6.3 HPW working for pay on/off-campus. However, in this study, participants spent an average of about only 1.9 HPW on part-time jobs on/off-campus, and more than 80% reported that they did not spend any time on part-time jobs. One possible explanation for this phenomenon is that most Chinese parents are willing and able to provide tuition and living expenses for their children, especially as a result of the decades long one-child policy in China [38]. Additionally, to support students in their studies, the Chinese government and individual universities provide an allotted amount of scholarships and bursaries for students seeking financial aid [39]. In contrast with the 7 HPW that students in the Fosnacht study spent on co-curricular activities and volunteering/community service, volunteering was investigated separately in this study, and more than half (54.08%, 371/686) of the participants did not spend any time on volunteering. This may be due to a combination of reasons. First of all, Chinese medical undergraduates are required to reside in on-campus dormitories that are often situated away from the community. These same students also come from single-child families and may therefore lack a strong sense of social responsibility and volunteerism. Moreover, Chinese medical students have few opportunities to participate in volunteering services related to their field of study (such as public health publicity, health care, medical education, etc.) [40]. Despite China being the only nation with a decades-long one-child policy, the above observations may be useful for future consideration, as other countries in the world are also seeing decreased family sizes and fertility rates [41]. For example, according to the UK Office of National Statistics, 40% of families had only one dependent child in 2017 [42]. This also points to an area of focus for further examination because medical students’ professionalism and interpersonal communication skills are closely associated with participation in volunteering [43]. In addition, Fosnacht et al. also considered students’ time use in caring for dependents and commuting to campus, which were not considered in this study because medical undergraduates are required to reside in on-campus dormitories in China and are deterred from having dependents when they apply for medical school [44].

Previous studies have suggested that high self-efficacy may be associated with better academic performance [45–48]. For example, a study of undergraduates at Cornell University by Ballen et al. found that self-efficacy could drive their performance gains with active learning [45]. Other researches have shown that the quantity of time in which students actively engage in studying is generally positively correlated with learning outcomes and academic success [12,18]. In this study, higher self-efficacy was associated with more time spent preparing for class and studying, which may establish a potentially effective pathway between self-efficacy and academic development. This means that self-efficacy may increase medical students’ time use in preparing for class and studying and thereby promote their academic performance. Bandura’s social cognitive theory could well explain a potential pathway between self-efficacy, time spent on studying, and academic performance among medical students [48]. Students with a strong sense of self-efficacy generally set higher academic goals and devote more time and energy to regulate their own learning in order to achieve their aspirations and academic accomplishments. Meanwhile, inverse causality between self-efficacy and time spent on studying cannot be excluded. That is, students with more time spent studying tend to gain stronger self-efficacy. Studies have shown that the most effective way individuals develop a strong sense of efficacy is through mastery experiences [49]. When students achieve their academic success by overcoming obstacles through perseverance, such as spending more time studying or improving studying efficiency, they would get a stronger and more resilient sense of self-efficacy. This process forms a positive cycle, where students with a strong sense of self-efficacy devote more time and energy to preparing for class and studying and achieve greater academic success, which in turn causes them to develop an even greater sense of self-efficacy. However, this study did not examine learning outcomes or academic performance, and further research on whether or not there are associations between self-efficacy, time spent studying, and academic performance are warranted.

Higher perceived stress was linked with the likelihood of medical students’ spending less time on preparing for class and studying, physical exercise, and volunteering. When reporting on the relationship between medical students’ participation in extracurricular activities and perceived stress, Fares et al. found that participation in physical exercise was shown to be associated with better stress outcomes, and participation in music-related activities were significantly correlated with better burnout outcomes among preclinical medical students [50]. One possible explanation for medical students with higher perceived stress levels spending less time on extracurricular activities is that medical students under stress may be more prone to burnout, characterized by emotional exhaustion, increased depersonalization, and a diminished sense of personal accomplishment [51], which is experienced in half of medical students [52]. Burnout in medical school has a negative impact on students’ academic development and overall well-being [53]. Medical students in burnout gradually lose enthusiasm, have a negative attitude, and even have an indifference for their studies and life in general and may eventually deteriorate into a state of reduced socialization and stagnation of personal development [54]. Therefore, in order to reduce stress and burnout, stress prevention and wellness education programs for undergraduates should be put in place in medical schools [55]. Of course, we do not exclude the fact that less time on preparing for class and studying, physical exercise, or volunteering may also lead to higher perceived stress. Physical exercise can be taken as an example. A systematic review revealed evidence that physical exercise results in less perceived stress [56]. This should not come as a surprise, since some individuals use exercise to cope with stress. Further research would be needed to illuminate the causal relationship between perceived stress and time use on preparing for class and studying, physical exercise, and volunteering among medical students.

It is worth noting that the majority (73.03%, 501/686) of medical students participating in this study reported that they spent less than 5 HPW participating in physical exercise, and 11.22% (77/686) of them reported that they never spent any time on physical exercise. Medical students make up the current pool of potential physicians and will eventually shoulder the important duties of curing diseases and saving lives, all of which require considerable physical and mental fitness. Since physical exercise has been proven to have positive impacts on both physical and mental health [57,58], medical schools are encouraged to take measures to promote medical students’ involvement in physical exercise. The results of this study demonstrated that with higher perceived stress, less time was devoted to physical exercise among medical students with a lower level of self-efficacy (Figure 1). In other words, self-efficacy may have moderated the negative association between perceived stress and time spent on physical exercise to some extent. This implies that improving medical students’ self-efficacy could be an effective measure to foster participation in physical activities, which would then enhance both physical and mental wellbeing.

### Limitations

This cross-sectional study was not able to identify the causal relationship between time use in out-of-class activities and self-efficacy and perceived stress. Despite the large sample size, this study only included second-year medical students from one medical university in China, and therefore conclusions may not be generalizable to all medical students in China. Additionally, this study only investigated the amount of time used in out-of-class activities and not the efficiency of time use or students’ time management skills. Longitudinal studies with greater representation and more accurate measures would be required to assess causality between time use in out-of-class activities, self-efficacy, and perceived stress and to provide a solid foundation for strategies aimed at developing medical students’ time management skills.

## Conclusion

In terms of time distribution in medical school, Chinese medical students may be spending more of their time preparing for class and studying and less time on volunteering and part-time jobs on/off-campus. Time spent on preparing for class and studying was positively associated with self-efficacy and negatively associated with perceived stress, which was also negatively associated with time volunteering and time doing physical exercise. Therefore, developing self-efficacy and maintaining a relatively low level of perceived stress, especially among students with lower self-efficacy, would be beneficial to medical students’ undergraduate experience.

## Supplementary Material

Supplemental Material

## Data Availability

The datasets used and/or analyzed in the present study are available from the corresponding author on reasonable request.
