# Supplementary material for: Time use in out-of-class activities and its association with self-efficacy and perceived stress: data from second-year medical students in China
Source: Med Educ Online. 2020 May 18;25(1):1759868. doi: 10.1080/10872981.2020.1759868 (PMC7301707; doi:10.1080/10872981.2020.1759868)
Supplement: Supplemental Material [file ZMEO_A_1759868_SM7450.docx]

**Supplementary** **Table S1** Associations between time spent on preparing for class and studying and self-efficacy and perceived stress using multivariate ordinal logistic regression (n=686)

| variable | Preparing for class and studying, OR(95%CI) | | | |
| --- | --- | --- | --- | --- |
|  | Model 1^a^ | Model 2^b^ | Model 3^c^ | Model 4^d^ |
| Self-efficacy | **1.40(1.22-1.62)***** | — | **1.29(1.10-1.52)**** | **1.31(1.11-1.53)**** |
| Perceived stress | — | **0.75(0.65-0.86)***** | **0.84(0.72-0.98)*** | **0.84(0.71-0.98)*** |
| Self-efficacy × Perceived stress | — | — | — | 1.04(0.93-1.16) |
| Age | **0.79(0.66-0.96)*** | **0.78(0.65-0.94)*** | **0.79(0.65-0.96)*** | **0.79(0.65-0.96)*** |
| Sex |  |  |  |  |
| Male(ref) | 1.00 | 1.00 | 1.00 | 1.00 |
| Female | 1.17(0.88-1.54) | 1.19(0.90-1.57) | 1.18(0.89-1.56) | 1.18(0.89-1.55) |
| Residence registration area |  |  |  |  |
| Village(ref) | 1.00 | 1.00 | 1.00 | 1.00 |
| City | 1.12(0.77-1.61) | 1.07(0.74-1.54) | 1.09(0.76-1.57) | 1.09(0.75-1.57) |
| Birthplace |  |  |  |  |
| Liaoning province(ref) | 1.00 | 1.00 | 1.00 | 1.00 |
| Other provinces | 1.15(0.86-1.52) | 1.17(0.88-1.55) | 1.14(0.86-1.52) | 1.15(0.86-1.53) |
| Medical school year of study |  |  |  |  |
| Five years(ref) | 1.00 | 1.00 | 1.00 | 1.00 |
| Eight years | 1.28(0.93-1.76) | 1.30(0.95-1.79) | 1.32(0.96-1.81) | 1.31(0.95-1.80) |
| Mother's education level |  |  |  |  |
| No high school(ref) | 1.00 | 1.00 | 1.00 | 1.00 |
| High school | 0.73(0.50-1.07) | 0.77(0.53-1.13) | 0.74(0.51-1.09) | 0.74(0.51-1.09) |
| Post-secondary or higher | 1.01(0.65-1.59) | 1.11(0.71-1.75) | 1.03(0.66-1.63) | 1.02(0.65-1.61) |
| Mother's Occupation |  |  |  |  |
| Legislators, senior officials, and managers(ref) | 1.00 | 1.00 | 1.00 | 1.00 |
| Professionals^1^ | 1.03(0.61-1.74) | 1.04(0.62-1.77) | 1.04(0.62-1.76) | 1.06(0.63-1.80) |
| Clerical support workers | 1.30(0.74-2.30) | 1.32(0.75-2.33) | 1.33(0.75-2.36) | 1.33(0.75-2.36) |
| Service and sales workers | 1.03(0.61-1.76) | 1.11(0.65-1.89) | 1.03(0.60-1.77) | 1.04(0.61-1.77) |
| Skilled agricultural, forestry, and fishery workers | 0.94(0.49-1.80) | 0.95(0.49-1.84) | 0.94(0.49-1.81) | 0.94(0.49-1.80) |
| Craft and related trades workers^2^ | **2.48(1.04-5.90)*** | **2.73(1.14-6.53)*** | **2.55(1.07-6.07)*** | **2.55(1.07-6.06)*** |
| Other | 0.88(0.53-1.45) | 0.88(0.53-1.46) | 0.88(0.53-1.45) | 0.88(0.53-1.45) |
| Annual household income |  |  |  |  |
| <¥20,000(ref) | 1.00 | 1.00 | 1.00 | 1.00 |
| ¥20,000-¥50,000 | 1.66(0.92-3.01) | 1.59(0.88-2.88) | 1.62(0.90-2.93) | 1.63(0.90-2.95) |
| ¥50,000-¥100,000 | 1.19(0.69-2.04) | 1.16(0.68-1.99) | 1.16(0.68-1.99) | 1.16(0.68-2.00) |
| >¥100,000 | 1.34(0.79-2.28) | 1.22(0.71-2.07) | 1.26(0.74-2.14) | 1.25(0.73-2.13) |

Note: *****p< .05; ******p< .01; *******p< .001. ^a^ Only self-efficacy was included; ^b^ Only perceived stress was included; ^c^ Self-efficacy and perceived stress were both included; ^d^ Self-efficacy, perceived stress and their interaction term were included. ^1^ Professionals, technicians, and associate professionals; ^2^ Craft and related trades workers, plant and machine operators and assemblers.

**Supplementary** **Table S2** Associations between time spent on leisure and recreation and self-efficacy and perceived stress using multivariate ordinal logistic regression (n=686)

| variable | Leisure and recreation, OR(95%CI) | | | |
| --- | --- | --- | --- | --- |
|  | Model 1^a^ | Model 2^b^ | Model 3^c^ | Model 4^d^ |
| Self-efficacy | 1.03(0.90-1.18) | — | 1.08(0.93-1.27) | 1.10(0.94-1.29) |
| Perceived stress | — | 1.08(0.93-1.24) | 1.12(0.95-1.32) | 1.12(0.95-1.31) |
| Self-efficacy × Perceived stress | — | — | — | 1.06(0.95-1.18) |
| Sex |  |  |  |  |
| Male(ref) | 1.00 | 1.00 | 1.00 | 1.00 |
| Female | 1.07(0.81-1.41) | 1.07(0.81-1.42) | 1.07(0.81-1.41) | 1.07(0.81-1.41) |
| Age | 0.83(0.68-1.01) | 0.83(0.68-1.01) | 0.83(0.68-1.01) | 0.83(0.68-1.01) |
| Residence |  |  |  |  |
| Village(ref) | 1.00 | 1.00 | 1.00 | 1.00 |
| City | 1.08(0.75-1.57) | 1.08(0.74-1.56) | 1.09(0.75-1.58) | 1.07(0.73-1.55) |
| Birthplace |  |  |  |  |
| Liaoning province(ref) | 1.00 | 1.00 | 1.00 | 1.00 |
| Other provinces | 1.07(0.81-1.41) | 1.08(0.81-1.43) | 1.07(0.81-1.42) | 1.07(0.81-1.42) |
| Medical school year of study |  |  |  |  |
| Five years(ref) | 1.00 | 1.00 | 1.00 | 1.00 |
| Eight years | 1.38(1.00-1.90) | 1.35(0.98-1.86) | 1.36(0.98-1.87) | 1.35(0.98-1.87) |
| Mother's education level |  |  |  |  |
| No high school(ref) | 1.00 | 1.00 | 1.00 | 1.00 |
| High school | 1.01(0.69-1.49) | 1.01(0.68-1.49) | 1.00(0.68-1.48) | 1.00(0.67-1.47) |
| Post-secondary or higher | 0.98(0.62-1.55) | 1.00(0.63-1.56) | 0.97(0.62-1.53) | 0.96(0.61-1.51) |
| Mother's Occupation |  |  |  |  |
| Legislators, senior officials, and managers(ref) | 1.00 | 1.00 | 1.00 | 1.00 |
| Professionals^1^ | 1.17(0.70-1.95) | 1.16(0.69-1.93) | 1.16(0.69-1.94) | 1.19(0.71-2.00) |
| Clerical support workers | 1.22(0.69-2.15) | 1.19(0.68-2.11) | 1.2(0.68-2.11) | 1.19(0.68-2.11) |
| Service and sales workers | 1.13(0.68-1.91) | 1.16(0.69-1.94) | 1.13(0.67-1.90) | 1.14(0.68-1.92) |
| Skilled agricultural, forestry, and fishery workers | 1.04(0.55-1.97) | 1.05(0.56-1.99) | 1.05(0.55-1.99) | 1.05(0.56-2.00) |
| Craft and related trades workers^2^ | 3.04(1.29-7.14) | 3.04(1.30-7.10) | 2.96(1.26-6.92) | 2.96(1.26-6.95) |
| Soldiers and other personnel | 1.40(0.86-2.29) | 1.40(0.86-2.28) | 1.39(0.85-2.27) | 1.40(0.86-2.28) |
| Annual household income |  |  |  |  |
| <¥20,000(ref) | 1.00 | 1.00 | 1.00 | 1.00 |
| ¥20,000-¥50,000 | 1.08(0.59-1.96) | 1.09(0.60-1.98) | 1.10(0.60-2.00) | 1.11(0.61-2.03) |
| ¥50,000-¥100,000 | 1.33(0.77-2.28) | 1.35(0.78-2.32) | 1.35(0.79-2.32) | 1.36(0.79-2.34) |
| >¥100,000 | **1.79(1.05-3.05)*** | **1.86(1.09-3.16)*** | **1.87(1.09-3.18)*** | **1.86(1.09-3.18)*** |

Note: *****p< .05; ******p< .01; *******p< .001. ^a^ Only self-efficacy was included; ^b^ Only perceived stress was included; ^c^ Self-efficacy and perceived stress were both included; ^d^ Self-efficacy, perceived stress and their interaction term were included. ^1^ Professionals, technicians, and associate professionals; ^2^ Craft and related trades workers, plant and machine operators and assemblers.

**Supplementary** **Table S3** Associations between time spent on physical exercise and self-efficacy and perceived stress using multivariate ordinal logistic regression (n=686)

| variable | Physical exercise, OR(95%CI) | | | |
| --- | --- | --- | --- | --- |
|  | Model 1^a^ | Model 2^b^ | Model 3^c^ | Model 4^d^ |
| Self-efficacy | 1.14(0.97-1.33) | — | 1.04(0.87-1.23) | 1.10(0.93-1.31) |
| Perceived stress | — | **0.80(0.68-0.93)**** | **0.81(0.68-0.96)*** | **0.82(0.69-0.97)*** |
| Self-efficacy × Perceived stress | — | — | — | **1.17(1.04-1.31)**** |
| Sex |  |  |  |  |
| Male(ref) | 1.00 | 1.00 | 1.00 | 1.00 |
| Female | **0.43(0.31-0.60)***** | **0.44(0.32-0.60)***** | **0.44(0.32-0.60)***** | **0.44(0.32-0.60)***** |
| Age | 1.04(0.84-1.30) | 1.04(0.83-1.29) | 1.04(0.83-1.29) | 1.05(0.84-1.30) |
| Residence |  |  |  |  |
| Village(ref) | 1.00 | 1.00 | 1.00 | 1.00 |
| City | 1.06(0.70-1.60) | 1.05(0.69-1.59) | 1.05(0.69-1.59) | 1.02(0.67-1.55) |
| Birthplace |  |  |  |  |
| Liaoning province(ref) | 1.00 | 1.00 | 1.00 | 1.00 |
| Other provinces | 0.94(0.68-1.29) | 0.93(0.67-1.28) | 0.92(0.67-1.27) | 0.93(0.68-1.28) |
| Medical school year of study |  |  |  |  |
| Five years(ref) | 1.00 | 1.00 | 1.00 | 1.00 |
| Eight years | **0.57(0.37-0.87)*** | **0.59(0.39-0.90)*** | **0.59(0.39-0.90)*** | **0.59(0.38-0.90)*** |
| Mother's education level |  |  |  |  |
| No high school(ref) | 1.00 | 1.00 | 1.00 | 1.00 |
| High school | 0.90(0.58-1.40) | 0.92(0.59-1.43) | 0.92(0.59-1.43) | 0.92(0.59-1.43) |
| Post-secondary or higher | 1.12(0.67-1.89) | 1.16(0.69-1.94) | 1.15(0.68-1.93) | 1.09(0.65-1.83) |
| Mother's Occupation |  |  |  |  |
| Legislators, senior officials, and managers(ref) | 1.00 | 1.00 | 1.00 | 1.00 |
| Professionals^1^ | 1.14(0.64-2.04) | 1.17(0.65-2.10) | 1.17(0.65-2.10) | 1.27(0.71-2.29) |
| Clerical support workers | 1.14(0.61-2.15) | 1.16(0.62-2.17) | 1.17(0.62-2.19) | 1.13(0.60-2.12) |
| Service and sales workers | 1.22(0.67-2.22) | 1.23(0.68-2.25) | 1.22(0.67-2.24) | 1.23(0.67-2.24) |
| Skilled agricultural, forestry, and fishery workers | 1.01(0.49-2.09) | 1.04(0.51-2.15) | 1.04(0.51-2.15) | 1.01(0.49-2.08) |
| Craft and related trades workers^2^ | 0.96(0.34-2.68) | 1.02(0.36-2.86) | 1.01(0.36-2.83) | 0.99(0.36-2.76) |
| Soldiers and other personnel | 1.00(0.57-1.75) | 1.02(0.59-1.79) | 1.03(0.59-1.80) | 1.01(0.57-1.76) |
| Annual household income |  |  |  |  |
| <¥20,000(ref) | 1.00 | 1.00 | 1.00 | 1.00 |
| ¥20,000-¥50,000 | 1.10(0.70-1.74) | 1.10(0.70-1.75) | 1.10(0.69-1.74) | 1.11(0.70-1.76) |
| ¥50,000-¥100,000 | 1.29(0.84-1.98) | 1.25(0.81-1.92) | 1.25(0.81-1.92) | 1.23(0.80-1.89) |
| >¥100,000 | 1.65(0.74-3.65) | 1.62(0.73-3.58) | 1.62(0.73-3.59) | 1.62(0.73-3.60) |

Note: *****p< .05; ******p< .01; *******p< .001. ^a^ Only self-efficacy was included; ^b^ Only perceived stress was included; ^c^ Self-efficacy and perceived stress were both included; ^d^ Self-efficacy, perceived stress and their interaction term were included. ^1^ Professionals, technicians, and associate professionals; ^2^ Craft and related trades workers, plant and machine operators and assemblers.

**Supplementary** **Table S4** Associations between time spent on student clubs and self-efficacy and perceived stress using multivariate ordinal logistic regression (n=686)

| variable | Student clubs, OR(95%CI) | | | |
| --- | --- | --- | --- | --- |
|  | Model 1^a^ | Model 2^b^ | Model 3^c^ | Model 4^d^ |
| Self-efficacy | 1.11(0.94-1.31) | — | 1.03(0.85-1.24) | 1.06(0.87-1.29) |
| Perceived stress | — | **0.84(0.71-0.99)*** | 0.85(0.71-1.03) | 0.85(0.71-1.03) |
| Self-efficacy × Perceived stress | — | — | — | 1.10(0.97-1.24) |
| Sex |  |  |  |  |
| Male(ref) | 1.00 | 1.00 | 1.00 | 1.00 |
| Female | 0.73(0.52-1.02) | 0.73(0.52-1.02) | 0.73(0.52-1.02) | 0.73(0.52-1.02) |
| Age | 1.00(0.80-1.26) | 1.00(0.79-1.25) | 1.00(0.79-1.26) | 1.01(0.80-1.27) |
| Residence |  |  |  |  |
| Village(ref) | 1.00 | 1.00 | 1.00 | 1.00 |
| City | 1.32(0.85-2.05) | 1.30(0.84-2.02) | 1.31(0.84-2.03) | 1.29(0.83-2.00) |
| Birthplace |  |  |  |  |
| Liaoning province(ref) | 1.00 | 1.00 | 1.00 | 1.00 |
| Other provinces | 1.05(0.75-1.48) | 1.05(0.75-1.47) | 1.05(0.75-1.47) | 1.05(0.75-1.48) |
| Medical school year of study |  |  |  |  |
| Five years(ref) | 1.00 | 1.00 | 1.00 | 1.00 |
| Eight years | 0.73(0.46-1.14) | 0.74(0.47-1.16) | 0.74(0.47-1.16) | 0.73(0.47-1.15) |
| Mother's education level |  |  |  |  |
| No high school(ref) | 1.00 | 1.00 | 1.00 | 1.00 |
| High school | 1.02(0.64-1.63) | 1.03(0.64-1.64) | 1.03(0.64-1.64) | 1.03(0.64-1.65) |
| Post-secondary or higher | 1.17(0.67-2.04) | 1.17(0.67-2.04) | 1.17(0.67-2.04) | 1.14(0.65-1.99) |
| Mother's Occupation |  |  |  |  |
| Legislators, senior officials, and managers(ref) | 1.00 | 1.00 | 1.00 | 1.00 |
| Professionals^1^ | 1.35(0.73-2.51) | 1.38(0.74-2.56) | 1.38(0.74-2.56) | 1.45(0.78-2.70) |
| Clerical support workers | 1.00(0.50-1.98) | 1.00(0.50-1.98) | 1.00(0.51-1.99) | 1.00(0.50-1.97) |
| Service and sales workers | 1.19(0.63-2.26) | 1.19(0.63-2.25) | 1.18(0.62-2.24) | 1.19(0.63-2.27) |
| Skilled agricultural, forestry, and fishery workers | 0.56(0.25-1.22) | 0.56(0.26-1.23) | 0.56(0.25-1.23) | 0.55(0.25-1.22) |
| Craft and related trades workers^2^ | 0.43(0.15-1.24) | 0.45(0.16-1.29) | 0.44(0.15-1.28) | 0.45(0.15-1.28) |
| Soldiers and other personnel | 0.84(0.46-1.54) | 0.84(0.46-1.55) | 0.84(0.46-1.55) | 0.84(0.46-1.54) |
| Annual household income |  |  |  |  |
| <¥20,000(ref) | 1.00 | 1.00 | 1.00 | 1.00 |
| ¥20,000-¥50,000 | 1.00(0.61-1.62) | 0.99(0.61-1.61) | 0.99(0.61-1.61) | 0.99(0.61-1.61) |
| ¥50,000-¥100,000 | **1.65(1.03-2.62)*** | **1.60(1.00-2.55)*** | **1.60(1.00-2.55)*** | **1.58(0.99-2.52)*** |
| >¥100,000 | 0.57(0.24-1.32) | 0.55(0.24-1.28) | 0.55(0.24-1.29) | 0.54(0.23-1.27) |

Note: *****p< .05; ******p< .01; *******p< .001. ^a^ Only self-efficacy was included; ^b^ Only perceived stress was included; ^c^ Self-efficacy and perceived stress were both included; ^d^ Self-efficacy, perceived stress and their interaction term were included. ^1^ Professionals, technicians, and associate professionals; ^2^ Craft and related trades workers, plant and machine operators and assemblers.

**Supplementary** **Table S5** Associations between time spent on volunteering and self-efficacy and perceived stress using binary logistic regression (n=686)

| variable | Volunteering, OR(95%CI) | | | |
| --- | --- | --- | --- | --- |
|  | Model 1^a^ | Model 2^b^ | Model 3^c^ | Model 4^d^ |
| Self-efficacy | **1.26(1.07-1.48)*** | — | 1.15(0.96-1.37) | 1.19(0.99-1.43) |
| Perceived stress | — | **0.76(0.65-0.90)**** | **0.81(0.67-0.97)*** | **0.80(0.67-0.97)*** |
| Self-efficacy × Perceived stress | — | — | — | 1.10(0.97-1.24) |
| Sex |  |  |  |  |
| Male(ref) | 1.00 | 1.00 | 1.00 | 1.00 |
| Female | **1.45(1.05-2.01)*** | **1.48(1.07-2.05)*** | **1.47(1.06-2.05)*** | **1.48(1.07-2.06)*** |
| Age | 1.06(0.85-1.33) | 1.06(0.85-1.32) | 1.06(0.85-1.33) | 1.07(0.86-1.34) |
| Residence |  |  |  |  |
| Village(ref) | 1.00 | 1.00 | 1.00 | 1.00 |
| City | 1.48(0.97-2.28) | 1.43(0.93-2.19) | 1.45(0.94-2.24) | 1.44(0.93-2.21) |
| Birthplace |  |  |  |  |
| Liaoning province(ref) | 1.00 | 1.00 | 1.00 | 1.00 |
| Other provinces | 1.04(0.75-1.44) | 1.04(0.75-1.44) | 1.03(0.74-1.43) | 1.03(0.74-1.44) |
| Medical school year of study |  |  |  |  |
| Five years(ref) | 1.00 | 1.00 | 1.00 | 1.00 |
| Eight years | **0.60(0.39-0.93)*** | **0.62(0.40-0.96)*** | **0.62(0.40-0.96)*** | **0.61(0.39-0.96)*** |
| Mother's education level |  |  |  |  |
| No high school(ref) | 1.00 | 1.00 | 1.00 | 1.00 |
| High school | 1.02(0.65-1.61) | 1.04(0.66-1.64) | 1.03(0.65-1.63) | 1.02(0.65-1.61) |
| Post-secondary or higher | 1.36(0.79-2.35) | 1.44(0.83-2.48) | 1.38(0.80-2.39) | 1.35(0.78-2.33) |
| Mother's Occupation |  |  |  |  |
| Legislators, senior officials, and managers(ref) | 1.00 | 1.00 | 1.00 | 1.00 |
| Professionals^1^ | 0.96(0.53-1.76) | 1.00(0.55-1.82) | 0.99(0.54-1.82) | 1.05(0.57-1.93) |
| Clerical support workers | 0.76(0.39-1.49) | 0.77(0.39-1.50) | 0.78(0.40-1.53) | 0.77(0.39-1.51) |
| Service and sales workers | 1.03(0.55-1.93) | 1.08(0.58-2.03) | 1.05(0.56-1.97) | 1.06(0.56-1.99) |
| Skilled agricultural, forestry, and fishery workers | 0.96(0.45-2.03) | 1.01(0.48-2.15) | 0.99(0.47-2.10) | 0.98(0.46-2.09) |
| Craft and related trades workers^2^ | 0.64(0.23-1.79) | 0.72(0.26-2.02) | 0.68(0.24-1.92) | 0.67(0.24-1.89) |
| Soldiers and other personnel | 1.35(0.75-2.41) | 1.40(0.78-2.52) | 1.39(0.77-2.50) | 1.39(0.77-2.50) |
| Annual household income |  |  |  |  |
| <¥20,000(ref) | 1.00 | 1.00 | 1.00 | 1.00 |
| ¥20,000-¥50,000 | 0.74(0.47-1.16) | 0.74(0.47-1.16) | 0.73(0.46-1.15) | 0.73(0.46-1.16) |
| ¥50,000-¥100,000 | 0.82(0.53-1.27) | 0.79(0.51-1.22) | 0.79(0.51-1.23) | 0.78(0.50-1.21) |
| >¥100,000 | **0.39(0.15-0.97)*** | **0.37(0.15-0.94)*** | **0.38(0.15-0.95)*** | **0.38(0.15-0.95)*** |

Note: *****p< .05; ******p< .01; *******p< .001. ^a^ Only self-efficacy was included; ^b^ Only perceived stress was included; ^c^ Self-efficacy and perceived stress were both included; ^d^ Self-efficacy, perceived stress and their interaction term were included. ^1^ Professionals, technicians, and associate professionals; ^2^ Craft and related trades workers, plant and machine operators and assemblers.

**Supplementary** **Table S6** Associations between time spend on part-time jobs on campus and self-efficacy and perceived stress using binary logistic regression (n=686)

| variable | Part-time job on campus, OR(95%CI) | | | |
| --- | --- | --- | --- | --- |
|  | Model 1^a^ | Model 2^b^ | Model 3^c^ | Model 4^d^ |
| Self-efficacy | 0.87(0.70-1.07) | — | 0.88(0.7-1.11) | 0.91(0.72-1.16) |
| Perceived stress | — | 1.09(0.89-1.35) | 1.04(0.82-1.31) | 1.06(0.84-1.33) |
| Self-efficacy × Perceived stress | — | — | — | 1.10(0.93-1.29) |
| Sex |  |  |  |  |
| Male(ref) | 1.00 | 1.00 | 1.00 | 1.00 |
| Female | 0.69(0.46-1.06) | 0.69(0.46-1.06) | 0.69(0.45-1.06) | 0.7(0.46-1.07) |
| Age | **0.73(0.55-0.98)*** | **0.73(0.55-0.98)*** | **0.73(0.55-0.98)*** | **0.74(0.55-0.99)*** |
| Residence |  |  |  |  |
| Village(ref) | 1.00 | 1.00 | 1.00 | 1.00 |
| City | **1.77(1.04-2.99)*** | **1.77(1.04-2.99)*** | **1.78(1.05-3.01)*** | **1.76(1.04-2.99)*** |
| Birthplace |  |  |  |  |
| Liaoning province(ref) | 1.00 | 1.00 | 1.00 | 1.00 |
| Other provinces | 1.07(0.69-1.65) | 1.07(0.69-1.65) | 1.07(0.69-1.66) | 1.08(0.7-1.67) |
| Medical school year of study |  |  |  |  |
| Five years(ref) | 1.00 | 1.00 | 1.00 | 1.00 |
| Eight years | **0.41(0.23-0.73)**** | **0.41(0.23-0.73)**** | **0.40(0.22-0.73)**** | **0.40(0.22-0.73)**** |
| Mother's education level |  |  |  |  |
| No high school(ref) | 1.00 | 1.00 | 1.00 | 1.00 |
| High school | 0.60(0.33-1.08) | 0.60(0.33-1.08) | 0.60(0.33-1.08) | 0.59(0.32-1.07) |
| Post-secondary or higher | 0.80(0.39-1.63) | 0.80(0.39-1.63) | 0.80(0.39-1.64) | 0.78(0.38-1.60) |
| Mother's Occupation |  |  |  |  |
| Legislators, senior officials, and managers(ref) | 1.00 | 1.00 | 1.00 | 1.00 |
| Professionals^1^ | 0.93(0.39-2.20) | 0.93(0.39-2.2) | 0.92(0.39-2.19) | 0.98(0.41-2.34) |
| Clerical support workers | 1.04(0.41-2.62) | 1.04(0.41-2.62) | 1.04(0.41-2.62) | 1.03(0.41-2.60) |
| Service and sales workers | 1.13(0.48-2.67) | 1.13(0.48-2.67) | 1.13(0.48-2.67) | 1.15(0.49-2.71) |
| Skilled agricultural, forestry, and fishery workers | 0.97(0.37-2.54) | 0.97(0.37-2.54) | 0.96(0.36-2.53) | 0.95(0.36-2.51) |
| Craft and related trades workers^2^ | 0.22(0.03-1.90) | 0.22(0.03-1.9) | 0.22(0.03-1.88) | 0.22(0.03-1.89) |
| Soldiers and other personnel | 1.13(0.50-2.52) | 1.13(0.50-2.52) | 1.12(0.5-2.51) | 1.13(0.51-2.54) |
| Annual household income |  |  |  |  |
| <¥20,000(ref) | 1.00 | 1.00 | 1.00 | 1.00 |
| ¥20,000-¥50,000 | 0.92(0.43-1.97) | 0.92(0.43-1.97) | 0.92(0.43-1.97) | 0.94(0.44-2.03) |
| ¥50,000-¥100,000 | 0.58(0.28-1.18) | 0.58(0.28-1.18) | 0.58(0.28-1.19) | 0.59(0.29-1.20) |
| >¥100,000 | 0.58(0.29-1.17) | 0.58(0.29-1.17) | 0.59(0.29-1.18) | 0.59(0.29-1.18) |

Note: *****p< .05; ******p< .01; *******p< .001. ^a^ Only self-efficacy was included; ^b^ Only perceived stress was included; ^c^ Self-efficacy and perceived stress were both included; ^d^ Self-efficacy, perceived stress and their interaction term were included. ^1^ Professionals, technicians, and associate professionals; ^2^ Craft and related trades workers, plant and machine operators and assemblers.

**Supplementary** **Table S7** Associations between time spend on part-time jobs off campus and self-efficacy and perceived stress using binary logistic regression (n=686)

| variable | Part-time job off campus, OR(95%CI) | | | |
| --- | --- | --- | --- | --- |
|  | Model 1^a^ | Model 2^b^ | Model 3^c^ | Model 4^d^ |
| Self-efficacy | 0.83(0.65-1.06) | — | 0.85(0.65-1.11) | 0.86(0.65-1.13) |
| Perceived stress | — | 1.11(0.87-1.42) | 1.04(0.79-1.35) | 1.05(0.80-1.38) |
| Self-efficacy × Perceived stress | — | — | — | 1.05(0.87-1.26) |
| Sex |  |  |  |  |
| Male(ref) | 1.00 | 1.00 | 1.00 | 1.00 |
| Female | 0.69(0.43-1.11) | 0.70(0.43-1.12) | 0.69(0.43-1.11) | 0.70(0.43-1.12) |
| Age | 1.15(0.84-1.57) | 1.15(0.84-1.58) | 1.14(0.84-1.57) | 1.15(0.84-1.57) |
| Residence |  |  |  |  |
| Village(ref) | 1.00 | 1.00 | 1.00 | 1.00 |
| City | 1.75(0.95-3.24) | 1.78(0.96-3.30) | 1.76(0.95-3.26) | 1.74(0.94-3.23) |
| Birthplace |  |  |  |  |
| Liaoning province(ref) | 1.00 | 1.00 | 1.00 | 1.00 |
| Other provinces | 1.02(0.62-1.68) | 1.01(0.62-1.65) | 1.02(0.62-1.68) | 1.03(0.63-1.68) |
| Medical school year of study |  |  |  |  |
| Five years(ref) | 1.00 | 1.00 | 1.00 | 1.00 |
| Eight years | 0.91(0.51-1.61) | 0.90(0.50-1.60) | 0.90(0.51-1.60) | 0.90(0.51-1.60) |
| Mother's education level |  |  |  |  |
| No high school(ref) | 1.00 | 1.00 | 1.00 | 1.00 |
| High school | 0.96(0.49-1.86) | 0.95(0.49-1.84) | 0.96(0.49-1.86) | 0.95(0.49-1.85) |
| Post-secondary or higher | 1.34(0.60-3.00) | 1.27(0.57-2.84) | 1.34(0.60-3.01) | 1.32(0.59-2.97) |
| Mother's Occupation |  |  |  |  |
| Legislators, senior officials, and managers(ref) | 1.00 | 1.00 | 1.00 | 1.00 |
| Professionals^1^ | 0.37(0.12-1.09) | 0.37(0.12-1.09) | 0.37(0.12-1.09) | 0.38(0.13-1.13) |
| Clerical support workers | 0.67(0.25-1.84) | 0.69(0.25-1.88) | 0.67(0.25-1.83) | 0.67(0.25-1.83) |
| Service and sales workers | 1.20(0.50-2.87) | 1.15(0.48-2.74) | 1.20(0.50-2.87) | 1.20(0.50-2.88) |
| Skilled agricultural, forestry, and fishery workers | 0.62(0.21-1.80) | 0.60(0.21-1.75) | 0.62(0.21-1.79) | 0.61(0.21-1.79) |
| Craft and related trades workers^2^ | 0.26(0.03-2.30) | 0.24(0.03-2.12) | 0.26(0.03-2.27) | 0.26(0.03-2.28) |
| Soldiers and other personnel | 0.86(0.37-1.99) | 0.85(0.37-1.96) | 0.86(0.37-1.98) | 0.86(0.37-1.99) |
| Annual household income |  |  |  |  |
| <¥20,000(ref) | 1.00 | 1.00 | 1.00 | 1.00 |
| ¥20,000-¥50,000 | 1.72(0.67-4.40) | 1.70(0.67-4.36) | 1.71(0.67-4.40) | 1.73(0.67-4.43) |
| ¥50,000-¥100,000 | 1.16(0.47-2.84) | 1.14(0.47-2.79) | 1.16(0.47-2.84) | 1.16(0.48-2.85) |
| >¥100,000 | 0.97(0.40-2.35) | 0.97(0.40-2.37) | 0.97(0.40-2.37) | 0.97(0.40-2.36) |

Note: *****p< .05; ******p< .01; *******p< .001. ^a^ Only self-efficacy was included; ^b^ Only perceived stress was included; ^c^ Self-efficacy and perceived stress were both included; ^d^ Self-efficacy, perceived stress and their interaction term were included. ^1^ Professionals, technicians, and associate professionals; ^2^ Craft and related trades workers, plant and machine operators and assemblers.
